# The worst case scenario: Locomotor and collision demands of the longest periods of gameplay in professional rugby union

**DOI:** 10.1371/journal.pone.0177072

**Published:** 2017-05-16

**Authors:** Cillian Reardon, Daniel P. Tobin, Peter Tierney, Eamonn Delahunt

**Affiliations:** 1 Leinster Rugby, Belfield, Dublin, Republic of Ireland; 2 University College Dublin, Belfield, Dublin, Republic of Ireland; Johns Hopkins University Bloomberg School of Public Health, UNITED STATES

## Abstract

A number of studies have used global positioning systems (GPS) to report on positional differences in the physical game demands of rugby union both on an average and singular bout basis. However, the ability of these studies to report quantitative data is limited by a lack of validation of certain aspects of measurement by GPS micro-technology. Furthermore no study has analyzed the positional physical demands of the longest bouts of ball-in-play time in rugby union. The aim of the present study is to compare the demands of the single longest period of ball-in-play, termed “worst case scenario” (WCS) between positional groups, which have previously been reported to have distinguishable game demands. The results of this study indicate that WCS periods follow a similar sporadic pattern as average demands but are played at a far higher pace than previously reported for average game demands with average meters per minute of 116.8 m. The positional differences in running and collision activity previously reported are perpetuated within WCS periods. Backs covered greater total distances than forwards (318 m *vs* 289 m), carried out more high-speed running (11.1 m·min^-1^ *vs* 5.5 m·min^-1^) and achieved higher maximum velocities (MaxVel). Outside Backs achieved the highest MaxVel values (6.84 m·sec^-1^). Tight Five and Back Row forwards underwent significantly more collisions than Inside Back and Outside Backs (0.73 & 0.89 collisions·min^-1^ *vs* 0.28 & 0.41 collisions·min^-1^ respectively). The results of the present study provide information on the positional physical requirements of performance in prolonged periods involving multiple high intensity bursts of effort. Although the current state of GPS micro-technology as a measurement tool does not permit reporting of collision intensity or acceleration data, the combined use of video and GPS provides valuable information to the practitioner. This can be used to match and replicate game demands in training.

## Introduction

Rugby union gameplay is characterized by repeated high intensity collisions and running efforts interspersed with periods of low intensity activity and rest [1–6]. Since the professionalisation of the game in 1995, there has been a gradual increase in the intensity of gameplay and the physical fitness requirements of players [3–5]. Rugby union players require well developed aerobic and anaerobic fitness to accommodate the game’s sporadic high intensity nature [7]. Rugby union positions are distinguishable by average gameplay activity profile [3, 4, 7–9] and also within singular bouts of gameplay [5].

Several studies have reported on the average gameplay demands of rugby union [1–4, 6, 8, 10]. These studies report a consensus that the high intensity component of gameplay for all positions is multi-activity in nature, but that forwards engage in more collisions and backs perform more high intensity running efforts. Austin, Gabbett & Jenkins [5] reported positional differences in rugby union within repeated high intensity efforts (RHIE). RHIE were defined as three or more sprint or collision exertions during the same passage of gameplay with less than 21 seconds between each exertion. More intense RHIE were reported for front row and back row forwards compared to inside and outside backs, due to the forwards engaging more often in long duration high intensity activities such as rucks, mauls and scrums. Studies from rugby league have used the same definition of RHIE to profile positional differences in gameplay demands [11].

To date, no studies have reported on the locomotor and collision demands of entire bouts of continuous ball-in-play time in rugby codes and the differences in these demands at varying levels of competition. The single longest period of continuous ball-in-play time from a game, termed the “worst case scenario,” (WCS) is likely to be much longer in duration and incorporate more high intensity efforts [5] than the average 28–52 seconds reported for RHIE bouts by Austin and colleagues [5]. Profiling the positional running and collision demands during the WCS will provide practitioners with useful information on the activity profile of prolonged bouts of gameplay.

It has been demonstrated that the level of competition impacts the intensity of gameplay in rugby league [12, 13]. Higher general low and high speed running and collision demands have been reported when playing bottom 4 versus top 4 National Rugby League teams [13]. However, single ball-in-play periods involving RHIE were more physically demanding and more frequent against higher quality opposition [12, 13]. Although rugby union research comparing elite versus sub-elite players reports superior physical characteristics of higher level players [14–17], there is a dearth of research comparing the locomotor and collision demands of players within continuous periods of ball-in-play.

Gabbett et al. [18] reported that the majority of RHIE occur in proximity to one or other try-line. Indeed, Austin, Gabbett and Jenkins [5] reported that 70% of tries are scored in close proximity to a RHIE. Gabbett and Gahan [19] reported similar findings. In the context of the WCS period, higher levels of competition are likely to involve better team defensive attributes and better ball retention by the attacking side. This may increase the likelihood of long periods of ball-in-play and the duration of the WCS period in a match. Team performance during these bouts may form a significant component of the margin between winning and losing at higher levels of competition.

GPS micro-technology is widely used in elite rugby union. It is a valid and reliable method of quantifying the locomotor demands of rugby union [20]. Much of the existing research into gameplay demands of prolonged bouts in rugby codes has been conducted in rugby league [12, 13,18], a code both qualitatively and quantitatively different to rugby union [1–6, 12,13,18]. Furthermore, application of this research to rugby union is problematic in that GPS micro-technology units (accelerometers, magnetometers and gyroscopes) were used to code collisions, a measure which has yet to be validated in a rugby union context. Using GPS micro- technology to analyse rugby union demands, Venter et al. [21] reported impact counts as high as 858 per game for forwards and 830 per game for backs. Similarly, high impact counts were also reported by Cunniffe et al. [1]. These contrast the findings of Roberts et al. [4], who reported an average of 89 collisions per game for forwards and 24 for backs based on video analysis. Research by Reardon et al. [22]. questions the validity of GPS micro-technology to correctly code collisions in rugby un ion. Although accelerations are the key component of sprint performance in rugby union [8], research by Akenhead et al. [23] reported 10 Hz GPS to be compromised as a tool for quantifying high rates of acceleration even in a controlled environment. Video analysis of rugby union has previously been used to report on collisions [4,8]. Roberts and colleagues [4] reported an inter-operator difference of 6.6% when reporting times spent in various activities in field sports including collisions. Finally, the tendency of research in the area to characterise movement speeds by absolute zones causes a significant shift in the interpretation of locomotor demands when compared to individualised bands [9]. In the only study to investigate singular bouts in rugby union [5], time motion analysis was used with broad subjective definitions of movement categories.

The aim of our study was to combine GPS and video analysis to establish the locomotor and collision demands of the WCS by analysing the single longest bout of uninterrupted gameplay across distinguishable positional groups for a series of games. At the same time, our study aimed to determine the specifics of long-bout demands associated with two different competitions in European professional rugby union. Knowledge of the demands of long bouts is important because of their relevance in determining outcomes at the highest levels of competition. Our analysis of the WCS provides data on the single longest duration bout of ball-in-play time in two professional rugby union competitions. This will inform practitioners preparing players for the most demanding physical periods of rugby union competition.

## Methods

### Participants

Thirty-nine elite professional rugby union players from a Guinness Pro12 team volunteered to participate in the study. The study was approved by the University College Dublin Human Research Ethics Committee (LS-14-03-Delahunt). Furthermore, each participant signed an informed consent form approved by the University College Dublin Human Research Ethics Committee. The participants (age = 27.2 ± 3.9 years, body mass = 99.2 ± 24.4 kg, height = 1.85 ± 0.43 m) cumulatively provided 200 GPS files from 6 games in the European Rugby Championship (ERC) and 11 games in the Guinness Pro12 league. Each player provided at least one GPS file with the largest number of files provided by any one player being fourteen.

### Procedures

All matches took place between September 6^th^ 2014 and January 24th 2015 on a Friday, Saturday or Sunday and were played on eleven different grounds used by clubs participating in the ERC and Pro12 in Ireland, England, Scotland, Wales, France and Italy. The ERC can be considered the higher level of competition between the two as teams qualify for ERC by finishing high in domestic leagues including the Pro12. Each consenting player wore a GPS micro-technology unit (mass = 67 g, size = 50·90 mm) (10 Hz S5, Catapult Innovations, Scoresby, VIC, Australia) in a bespoke pocket fitted in his playing jersey on the upper thoracic spine between the scapulae. The GPS device captured data at a sampling frequency of 10 Hz. The reliability of the unit has previously been demonstrated as acceptable for measuring speed and distances in team sports [19,22,23]. All participants were familiarized with the devices as part of their day- to-day training and playing practices. Each player wore the same assigned GPS unit throughout the course of the data collection period.

The GPS units were switched on at least 10 minutes prior to the game to ensure a full high quality satellite signal. During each match, the real-time GPS data was monitored and cut into periods, each representing continuous bouts of ball-in-play time. Appropriate substitutions were also noted in the software enabling full knowledge of each player’s participation. The definition of a bouts duration was from the time the ball entered play until it went dead or until play was stopped by the referee. Following the game, GPS data was downloaded to a laptop and analyzed with Sprint 5.1 software (Catapult Innovations, Scoresby, VIC, Australia). Sprint software was used to identify the single longest bout (WCS) in each game. The GPS data file for each participating player from the WCS bout was downloaded with Sprint 5.1 and exported to Microsoft Excel (Microsoft Corporation, USA).

For the purposes of data analysis and comparisons with previous studies [5], players were assigned to a positional category, of which there were four. These positional groups have previously been reported to have distinctive average game demands [3,4,9] and within single bouts [5]. The positional sub-categories used were as follows: (1) Tight Five; (2) Back Row; (3) Inside Back; (4) Outside Back.

#### Locomotor variables

The total distance (m) covered in the WCS bout from each game and total distance relative to the bouts duration (m·min^-1^; MPM) was calculated for each data file. The maximum velocity (MaxVel) of each participant was established by analyzing all training and playing data throughout the previous two seasons. This included dedicated MaxVel training. Speed zones were individualized as percentages of each players MaxVel as per Reardon et al [9]. Speed zone classifications were as follows: Walk (< 2 m·s^-1^), low-speed running (2 m·s^-1^–59.9% MaxVel), high-speed running (≥ 60% MaxVel), sprint efforts (≥ 90% MaxVel of duration ≥ 0.2 sec).

#### Collision measurement

Post-game analyses was conducted by two expert video analysts to determine the number of collisions undergone by each player during the WCS period. Video analysis has been previously used to analyze collisions in rugby union match play [4,5,24]. The collision count was considered to be the count of all tackles scrums, mauls, carries into contact and positive impact rucks.

#### Statistical analysis

To investigate whether differences exist in the output of players from each positional category regardless of competition level a multivariate analysis of variance were performed. The independent variable was positional category [Tight Five Forwards; Back Row Forwards; Inside Backs; Outside Backs]. The dependent variables were: [1] Total distance; [2] MPM; [3] MaxVel; [4] Walk distance; [5] low-speed running (LSR); [6] high-speed running (HSR); [7] Sprint efforts; [8] Collisions.

To investigate the influence of level of competition on the output of players from each positional category, four separate multivariate analyses of variance were performed. In each case the independent variable was level of competition (ECC vs Pro12). [1] Total distance; [2] MPM; [3] MaxVel; [4] Walk distance; [5] low-speed running (LSR); [6] high-speed running (HSR); [7] Sprint efforts; [8] Collisions.

## Results

There was a statistically significant difference between the output of players from each positional category regardless of competition on the combined dependent variables, F (24, 548) = 36.87, p < 0.001, Wilk’s Lambda = 0.06, partial eta squared = 0.61. Details of the Bonferroni adjusted pairwise comparisons are outlined in Table 1.

**Table 1:** Locomotor and collision demands of each positional category.

	Tight Five Forwards	Back Row Forwards	Inside Backs	Outside Backs
Average Duration (s)	161	152	154	155
Total distance (m)	289 (272–305)	290(270–309)	318 (299–336)	319 (297–341)
MPM (m·min^-1^)	109 (104–114)^c^^,^^d^	111 (105–117)^c^^,^^d^	123 (117–129)^a^^,^^b^	124 (117–131)^a^^,^^b^
MaxVel (m·s^-1^)	4.9 (4.70–5.12)^b^^,^^c^^,^^d^	5.72 (5.48–5.97)^a^^,^^d^	6.02 (5.79–6.25)^a^^,^^d^	6.84 (6.57–7.12)^a^^,^^b^^,^^c^
Walk Distance (m·min^-1^)	45 (42–49)	40 (36–44)	43 (39–46)	47.71 (43–52)
LSR (m·min^-1^)	97 (89–104)^b^^,^^c^^,^^d^	65 (56–73)^a^	72 (64–80)^a^	62 (52–71)^a^
HSR (m·min^-1^)	4.9 (3–6.9)^d^	6.0 (3.8–8.3) ^d^	8.1 (6.0–10.2) ^d^	14.1 (11.6–16.7)^a^^,^^b^^,^^c^
Sprint Efforts	0.02 (-0.04–0.07)	0.02 (-0.04–0.08)	0.06 (0.00–0.11)	0.11 (0.04–0.16)
Collisions (min^-1^)	0.73 (0.62–0.84)^c^^,^^d^	0.89 (0.75–1.01)^c^^,^^d^	0.28 (0.17–0.40)^a^^,^^b^	0.41 (0.27–0.56) ^a^^,^^b^

Values are mean (95% CI). MPM = total distance relative to the bouts duration; MaxVel = maximum velocity; LSR = low-speed running; HSR = high-speed running.

^a^ = significantly different to Tight Five Forwards.

^b^ = significantly different to Back Row Forwards.

^c^ = significantly different to Inside Backs.

^d^ = significantly different to Outside Back.

Regarding the influence of level of competition on the output of players from each positional category, the results were as follows. For the Tight Five Forwards positional category there was a statistically significant main effect on the combined dependent variables, F (8, 54) = 2.06, p ≤ 0.05, Wilk’s Lambda = 0.76, partial eta squared = 0.23. Tight Five Forwards performed significantly more HSR during ECC games (8.87 m.min^-1^) compared to Pro12 games (3.18 m.min^-1^). Details of the Bonferroni adjusted pairwise comparisons are outlined in Table 2. For the Back Row Forwards positional category there was no statistically significant main effect on the combined dependent variables, F (8, 38) = 1.39, p ≥ 0.05, Wilk’s Lambda = 0.77, partial eta squared = 0.23 (Table 3). For the Inside Backs positional category there was no statistically significant main effect on the combined dependent variables, F (8, 44) = 1.01, p ≥ 0.05, Wilk’s Lambda = 0.85, partial eta squared = 0.15 (Table 4). For the Outside Backs positional category there was no statistically significant main effect on the combined dependent variables, F (8, 44) = 1.01, p ≥ 0.05, Wilk’s Lambda = 0.85, partial eta squared = 0.15 (Table 5).

**Table 2:** Tight Five Forwards: Locomotor and collision demands during ERC and Pro12.

	ERC	Pro12	Mean difference	95% CI of mean difference (lower bound)	95% CI of mean difference (upper bound)	Partial Eta Squared
Distance (m)	297(269–326)	285 (265–304)	12.54	-22.67	47.35	0.008
MPM (m·min^-1^)	111 (102–120)	108(102–114)	2.74	-8.05	13.53	0.004
MaxVel (m·s^-1^)	5.17 (4.82–5.51)	4.79 (4.56–5.03)	0.37	-0.41	0.79	0.051
Walk Distance (m·min^-1^)	48 (41–55)	45 (40–49)	3.73	-4.93	12.39	0.012
LSR (m·min^-1^)	89 (72–106)	100(89–112)	- 11.14	-31.65	9.37	0.019
HSR (m·min^-1^)	8.9 (5.8–11.9)	3.2 (1.1–5.3)^*^	5.69	1.99	9.38	0.135
Sprint Efforts	0.05 (-0.01–0.11)	0.00 (-0.04–0.04)	0.05	-0.02	0.12	0.035
Collisions (min^-1^)	0.77 (0.56–0.99)	0.71 (0.56–0.85)	0.067	-0.21	0.33	0.004

Values are mean (95% CI). ERC = European Rugby Championship; Pro12 = Guinness Pro12; MPM = total distance relative to the bouts duration; MaxVel = maximum velocity; LSR = low-speed running; HSR = high-speed running

^*^ = significantly different from ERC (p < 0.05).

**Table 3:** Back Row Forwards: Locomotor and collision demands during ERC and Pro12.

	ECC	Pro12	Mean difference	95% CI of mean difference (lower bound)	95% CI of mean difference (upper bound)	Partial Eta Squared
Distance (m)	287 (253–322)	291 (267–314)	-3.78	-45.53	37.98	0.001
MPM (m·min^-1^)	109 (99–119)	112 (105–119)	-3.32	-15.78	9.13	0.006
MaxVel (m·s^-1^)	5.69 (5.23–6.15)	5.74 (5.43–6.06)	-0.05	-0.61	0.50	0.001
Walk Distance (m·min^-1^)	37 (33–41)	42 (39–44)	-4.25	-8.96	0.46	0.068
LSR (m·min^-1^)	66 (55–78)	64 (57–72)	1.85	-11.87	15.58	0.002
HSR (m·min^-1^)	5.4 (2.4–8.5)	6.4(4.3–8.4)	-0.94	-4.64	2.75	0.006
Sprint Efforts	0.00 (-0.08–0.08)	0.03 (-0.02–0.08)	-0.03	-0.12	0.06	0.01
Collisions (min^-1^)	0.85 (0.56–1.15)	0.89 (0.69–1.09)	-0.04	-0.39	0.32	0.001

Values are mean (95% CI). ERC = European Rugby Championship; Pro12 = Guinness Pro12; MPM = total distance relative to the bouts duration; MaxVel = maximum velocity; LSR = low-speed running; HSR = high-speed running.

**Table 4:** Inside Backs: Locomotor and collision demands during ERC and Pro12.

	ECC	Pro12	Mean difference	95% CI of mean difference (lower bound)	95% CI of mean difference (upper bound)	Partial Eta Squared
Distance (m)	306 (275–337)	323 (302–343)	-16.44	-53.26	20.38	0.016
MPM (m·min^-1^)	118 (106–130	125 (118–133)	-7.42	-21.42	6.58	0.022
MaxVel (m·s^-1^)	6.05 (5.60–6.51)	6.01 (5.7–6.31)	0.05	-0.49	0.58	0.001
Walk Distance (m·min^-1^)	50 (43–57)	40 (36–45)	9.60	1.17	18.02	0.093
LSR (m·min^-1^)	59 (45–74)	77 (68–87)	-18.10	-35.49	-0.72	0.079
HSR (m·min^-1^)	8.9 (5.1–12.6)	7.7 (5.3–10.2)	-1.10	-3.38	5.58	0.005
Sprint Efforts	0.06 (-0.06–0.18)	0.05 (-0.02–0.13)	0.01	-0.13	0.15	0.000
Collisions (min^-1^)	0.34 (0.22–0.46)	0.26 (0.18–0.34)	0.08	-0.07	0.23	0.023

Values are mean (95% CI). ERC = European Rugby Championship; Pro12 = Guinness Pro12; MPM = total distance relative to the bouts duration; MaxVel = maximum velocity; LSR = low-speed running; HSR = high-speed running.

**Table 5:** Outside Backs: Locomotor and collision demands during ERC and Pro12.

	ECC	Pro12	Mean difference	95% CI of mean difference (lower bound)	95% CI of mean difference (upper bound)	Partial Eta Squared
Distance (m)	321 (270–371)	318 (286–351)	2.21	-57.72	62.15	0.000
MPM (m·min^-1^)	120 (106–133)	125 (117–134)	-5.68	-21.42	10.07	0.015
MaxVel (m·s^-1^)	6.36 (5.82–6.91)	7.05 (6.69–7.40)	-0.68	-1.34	-0.03	0.114
Walk Distance (m·min^-1^)	54 (43–64)	45 (38–52)	8.61	-3.68	20.90	0.055
LSR (m·min^-1^)	56 (43–69)	64 (56–73)	-7.92	-23.65	7.81	0.029
HSR (m·min^-1^)	9.7 (3.1–16.2)	16 (11.8–20.3)	-6.34	-14.18	1.43	0.073
Sprint Efforts	0.00 (-0.19–0.19)	0.15 (0.03–0.28)	-0.15	-0.38	0.07	0.051
Collisions (min^-1^)	0.35 (0.09–0.60)	0.44 (0.28–0.61)	-0.10	-0.40	0.21	0.012

Values are mean (95% CI). ERC = European Rugby Championship; Pro12 = Guinness Pro12; MPM = total distance relative to the bouts duration; MaxVel = maximum velocity; LSR = low-speed running; HSR = high-speed running.

## Discussion

The findings of this study support previous research reporting the intermittent nature of rugby union gameplay [3,4,8,25]. Within the WCS, the majority of activity is carried out at low intensity with intermittent bursts of high intensity collision and running activity. Additionally, the findings of this research show that the general intensity and pace of the WCS period to be far greater (average MPM = 117 m·min^-1^) than previously reported when analysing average game demands (average MPM = 68 m·min^-1^) [10].

Our study observed that within the WCS bouts, forward positions are characterized by both more low speed running and more collisions than back positions. Back Row Forwards produced higher MaxVel than Tight Five Forwards (5.7 m·s^-1^ *vs* 4.9 m·s^-1^) in WCS periods but carried out less LSR (65 m·min^-1^ *vs* 97 m·min^-1^). The Inside Backs and Outside Backs positional categories were characterized by higher MaxVel than Tight Five Forwards during WCS bouts, with Outside Backs producing the highest MaxVel (6.8m·s^-1^) and carrying out the most HSR (14.1 m·min^-1^). This is consistent with research on the global demands of rugby union which reports that the high intensity activity profile of forwards is more collision based while backs carry out more high intensity running and sprinting [3, 6, 8].

This study differs from existing research in rugby union gameplay demands in its reporting of sprint efforts. The average number of sprint efforts per WCS period across all positions was 0.03. Austin et al. [5] previously reported that 45% of the activity of Inside Backs and Outside Backs within RHIE was sprinting. Duthie et al. [25] reported an average of 11 sprints per game for Forwards and 27 for Backs. Roberts et al. [4] reported rugby union Forwards to produce 16 sprints per game compared to 23 for Backs.

It is likely that the discrepancy in sprint frequency reported between our study and that in the published literature arises from methodological differences. Austin et al. [5] and Duthie et al. [25] used time-motion analysis and subjective descriptions of movement categories to analyse rugby union demands. Roberts et al. [4] and Cunniffe et al. [1] used video and GPS analysis respectively as well as quantitative measures of movement categorisation. However, the speed thresholds applied in these reports (6.7 m·s^-1^ and 5.6 m·s^-1^ respectively) are much lower than those used in our research. Our study classified sprinting as being in excess of 90% MaxVel in accordance with individualised speed zones [9]. Our own training and match data shows that Backs regularly reach velocities of over 9 m·s^-1^ in sprints in excess of 40 m. This method of measurement makes the achievement of sprint speeds in this research much less likely than in any of the aforementioned research. One study [3] used GPS to evaluate distance covered at various velocities in international rugby union, reporting an average of 70 meters per game covered at >8 m·s^-1^ across all positions with the highest total sprint distance reported for the Wing position category; at 140 m per game. Although this is not a large component of total distance covered, the values represent a far greater sprint demand on players than is reported by our study for the WCS. Furthermore, the threshold of 8m·s is comparable to the 90% MaxVel sprint threshold employed by our study particularly in the case of the Inside Back and Outside Back position categories. It may be the case that because of the definition of WCS periods of play that the likelihood of incorporating sprints is reduced when compared to an average analysis of rugby union as per Quarrie et al. [3]. As the WCS is defined as the single longest period of continuous play from a game, it is likely that this period of gameplay be characterized by a pattern of “phases”. This type of structured game pattern would limit running distances and may account for the relatively low MaxVel values observed and lack of sprint efforts.

Previous research in rugby codes [12, 13] suggests that high intensity activity demands are greater when playing higher quality opposition and that anthropometric and athletic profiles of elite players are superior to those reported at sub-elite levels of competition [14–17]. Because teams qualify for the ERC by finishing high in their domestic leagues, it may be classified as a higher level of competition than the Pro12. However, our statistical analysis indicates very little difference in the physical demands of WCS periods between the two competitions. Only WCS demands for Tight Five Forwards differed significantly between Pro12 and ERC, whereby the HSR demands were higher in ERC (8.87 m·min^-1^ vs 3.18 m·min^-1^). This may be a reflection of the parameters of the WCS period. Alternatively, it may due to there not being a significant difference in physical demands between the two competitions with both ERC and Pro12 being elite professional competitions.

Despite a lack of statistically significant differences between competitions, our data reflects some inter-competition variance in position demands that appear to have practical significance. These differences would certainly influence a practitioner’s view of player preparation with respect to game demands. In the Pro12, Inside Backs and Outside Backs positions perform higher MPM versus the ERC (125 m·min^-1^ & 125 m·min^-1^ vs 118 m·min^-1^ & 120 m·min^-1^ respectively). This difference arises from a shift in movement mode from walking into LSR. Additionally, Outside Backs in this study produced more HSR in Pro12 versus ECC (16 m·min^-1^ vs 9.7 m·min^-1^). This indicates an increase in the pace of the game in the Pro12 competition for Inside and Outside backs. These findings are congruent with rugby league research [13], which reported greater running demands against weaker opposition, ostensibly due to increased availability of space. A simultaneous shift in collision patterns amongst Backs positions is observed between competitions in the current study. Outside backs perform more collisions in the Pro12 whereas Inside Backs collision count is higher in the ERC. This may be a commentary on the areas of the pitch in which contests for ball possession occur between competitions. Hypothetically, a more expansive running game played in the Pro12 would deliver more ball possession to the Outside Backs and result in more collisions in those positions. Conversely, a more structured pattern with greater intensity of ball contest would result in more collisions in the middle of the field combined with lower HSR demands in Backs positions. This is what we have observed in the data collected from ERC. This perspective on competition demands seems to be supported by the activity patterns observed for Tight Five Forwards in this study. The reported increase in high intensity running demands for this position in ERC versus Pro 12 combined with statistically non-significant increases in collisions in sprint efforts in the ERC indicate for a style of play more dominated by ball contesting activities and consequently by Forwards and Inside Backs positions. Although based predominantly around statistically non-significant differences, the observed variance in positional WCS activity patterns between competitions occurs with a degree of consistency that, when regarded as whole and combined with experiential evidence of professional players, coaching staff and analysts, constitutes a considerable argument for distinguishable patterns of gameplay between Pro12 & ERC.

Our research is limited in its inability to measure certain important aspects of WCS demands. The typical pattern of running in rugby union is one of repeated short sprints. Research consistently reports average sprint distances of 6–20 m in field sports including rugby union [26–28]. This makes acceleration the most important predictor of sprint performance and the number and intensities of accelerations being the driver of physiological damage associated with sprinting in rugby union. Currently GPS technology does not allow for accurate measurement of high rates of acceleration [23]. Currently GPS technology cannot accurately count collisions or quantify collision intensity in rugby union [23]. Subjectively, it is possible that not all collision activity is high intensity activity. It has been shown that Prop Forwards produce more force when scrummaging compared to Locks and Back Row Forwards [29]. The ability to measure the intensity of collisions is key to improving our understanding of the average and single bout demands of rugby union.

Using a combination of GPS and video analysis, a large sample size and individualised speed zones [9], our study makes a substantial contribution to the knowledge of the WCS demands in rugby union. Furthermore it provides information on the positional activity profile of WCS at two different levels of competition. Our research reports on differences in physical output during the longest bout of gameplay in two European professional rugby union competitions. Its use to the practitioner is that demands of training can be monitored and load management strategies devised which allow matching of training demands to WCS bouts. Our research does not provide insight into the characteristics of successful versus unsuccessful bouts. While our research has value in providing practitioners with general information around demanding bouts of play at different levels of competition, it is undoubtedly impacted by technical and tactical characteristics specific to the rugby union club used in the research, individualities of players within the subject group and features of the competitions analysed. In order to get information that is specific to other teams and competitions, practitioners should carry out similar research in their own contexts. Future research should centre on achieving a valid method of quantification of collision and acceleration forces. This would greatly improve the interpretation of rugby union demands on both an average and in-bout basis.
